# Pulsed Microwave Energy Transduction of Acoustic Phonon Related Brain Injury

**DOI:** 10.3389/fneur.2020.00753

**Published:** 2020-08-04

**Authors:** Graham K. Hubler, Stuart W. Hoffman, Tim D. Andreadis, Ralph G. DePalma

**Affiliations:** ^1^The School of Medicine, University of Missouri, Columbia, MO, United States; ^2^US Department of Veterans Affairs, Rehabilitation Research and Development Service, Office of Research and Development, Veterans Health Administration, Washington, DC, United States; ^3^U.S. Naval Research Laboratory, Tactical Electronic Warfare Division, Washington, DC, United States; ^4^US Department of Veterans Affairs, Office of Research and Development, Veterans Health Administration, Washington, DC, United States; ^5^Department of Surgery, Uniformed University of the Health Sciences, Bethesda, MD, United States

**Keywords:** microwave, phonon, U.S. embassy exposures, cognitive disorder, mTBI

## Abstract

Pulsed microwaves above specific energy thresholds have been reported to cause brain injury in animal models. The actual physical mechanism causing brain damage is unexplained while the clinical reality of these injuries remains controversial. Here we propose mechanisms by which pulsed microwaves may injure brain tissue by transduction of microwave energy into damaging acoustic phonons in brain water. We have shown that low intensity explosive blast waves likely initiate phonon excitations in brain tissues. Brain injury in this instance occurs at nanoscale subcellular levels as predicted by physical consideration of phonon interactions in brain water content. The phonon mechanism may also explain similarities between primary non-impact blast-induced mild Traumatic Brain Injury (mTBI) and recent clinical and imaging findings of unexplained brain injuries observed in US embassy personnel possibly due to directed radiofrequency radiation. We describe experiments to elucidate mechanisms, RF frequencies and power levels by which pulsed microwaves potentially injure brain tissue. Pathological documentation of nanoscale brain blast injury has been supported experimentally using transmission electron microscopy (TEM) demonstrating nanoscale cellular damage in the absence of gross or light microscopic findings. Similar studies are required to better define pulsed microwave brain injury. Based upon existing findings, clinical diagnosis of both low intensity blast and microwave-induced brain injury likely will require diffusion tensor imaging (DTI), a specialized water based magnetic resonance imaging (MRI) technique.

## Introduction

Swanson et al. (1) examined 24 US Cuban embassy personnel exposed to an unknown directed energy source. They found that 21 of those examined had clinical findings similar to mild traumatic brain injury (mTBI). All 24 individuals reported audible and sometimes painful sounds during possible exposures. Hoffer et al. (2) examined a partially overlapping group of 35 embassy-related individuals, among whom 25 reported auditory phenomena and post incident symptoms along with 10 individuals who lived with the affected persons who did not report hearing sounds. These workers found that all 25 individuals hearing sounds had vestibular abnormalities; over half of these exhibited cognitive disorders. The ten individuals who did not report hearing sounds did not exhibit vestibular or cognitive abnormalities. Verma et al. (3) expanded Swanson's 2018 report to include 40 government employees describing acoustic experiences. These individuals had neurological symptoms suggesting mTBI. Significant brain structural abnormalities were documented using advanced specialized MRI imaging of this cohort (3).

The method of delivery of damaging energy to these personnel remains controversial. A sonic source initially was postulated because subjects heard high-pitched sounds during the incidents (4–8). Lin (6, 7) suggested the mode of attack as a possible directed energy source of pulsed microwaves based on observations that pulsed microwaves are audible to those irradiated. Microwaves can also be focused into narrow field-of-view beams in order to target individuals.

Experimental evidence indicates that pulsed microwaves can induce disruption in brain tissue producing subsequent behavioral and cognitive dysfunction. Thomas et al. (9) early reported that pulsed microwaves disrupted acquisition performance in rats. Wang and Lai (10) later demonstrated that acute exposures to pulsed microwaves impaired reference memory in rats. In addition, pulsed microwaves reportedly may alter blood-brain barrier permeability, disrupt long-term potentiation and result in DNA strand breaks (11). Pakhomov and Murphy (12) reviewed an extensive body of microwave experiments performed in Russia and the former Union of Soviet Socialist Republics. In these works, animal brains were found to be considerably more sensitive to pulsed microwaves than to continuous wave microwaves; they concluded that microwave heating did not, at least primarily, cause this injury effect. Thermal sensors placed in rabbit brains showed no more than a 0.2°C temperature increase in animals displaying cognitive impairments.

Mechanisms by which pulsed microwave energy injure or impair the brain remain unclear. Based upon our prior physical considerations of low intensity primary blast and crystalline fracture effects (13, 14), we here describe physical mechanisms by which microwave energy may produce brain injuries similar to those caused by primary blast exposure. Using documented experimental physical findings, we consider the hypothesis that primary blast shock waves due to explosions and pulsed microwaves may both excite GHz frequency phonons in brain water content to cause nanoscale subcellular brain injuries.

## Phonon Model of Brain Injury: Ultrastructural Effects

Based upon observation of failure wave effects in brittle solids (13), Kucherov et al. (14) developed a primary brain blast injury hypothesis based on water behaving as a brittle solid under shock wave blast loading. Calculation of brain cellular injury dimensions were based upon the fact that the water content of brain tissue is 70–80% and that of cerebrospinal fluid 100%. They postulated that shock waves from an explosive blast excited high frequency THz phonons in brain water (14, 15). Energy stored in optical phonons decays within nanoseconds to lower frequency acoustic phonons creating damage when brain tissue strength is exceeded. A phonon bottleneck occurs where the 7.5 GHz phonon (lowest frequency acoustic phonon in water) decays to the ground state (16, 17). Energy stored from higher frequency phonons pumps the amplitude of the 7.5 GHz phonon until water molecules rupture to disrupt brain tissue. When phonon generated waves have sufficiently high amplitude, tissue across the peaks of the phonon wavelength will be sheared. The resulting wavelength can be used to estimate the dimensions of tissue and cellular damage. Based upon the speed of sound in water (1,500 m/s) and a phonon frequency of 7.5 GHz, phonon associated brain damage was predicted to occur at ~200 nm intervals (200 nm = 1,500 m/s ÷ 7.5 × 10^9^ cycles/s) at peaks of wave forms exceeding tissue strength (14). Injury dimensions would measure ~3–6 nm approximating the dimensions of cell membranes and other intracellular structures.

To test this hypothesis, Song et al. (18, 19) subjected mice to open field blasts using 350 g of C4 explosive. Mice were placed 2.1, 3, 5, and 7 meters from a blast source (18). Cognitive and behavioral testing showed that deficit severity correlated with closeness to the blast, overpressure and impulse exposure. Subsequent TEM of these brains showed nanoscale intracellular neuronal damage consistent with damage dimensions predicted by phonon injury assuming 100% water brain content (19). Shock damage occurs within microseconds as the shock wave passes though the brain at the speed of sound in water, in contrast to milliseconds required for inertial or impact injuries. Cellular damage occurs in well-documented absence of head motion at blast exposures approximating 47–87 kPa (18, 19). These observations support the hypothesis that 7.5 GHz acoustic phonons in brain water content likely explain nanoscale brain damage in non-impact low intensity blast exposures. Since gross and light microscopic changes are absent under these circumstances, detection of subcellular damage as a result of microwave exposure suggests use of TEM. Gross examination and conventional light microscopy should be supplemented by TEM. This is yet to be done for microwave brain injury. Similarly, as in low intensity blast exposures, diffusion tensor imaging (DTI), a water-based imaging technique, will likely be required to detect clinical microwave effects invisible to conventional imaging.

## Microwave Effects on Auditory System and Brain Tissue

**Figure 1:**
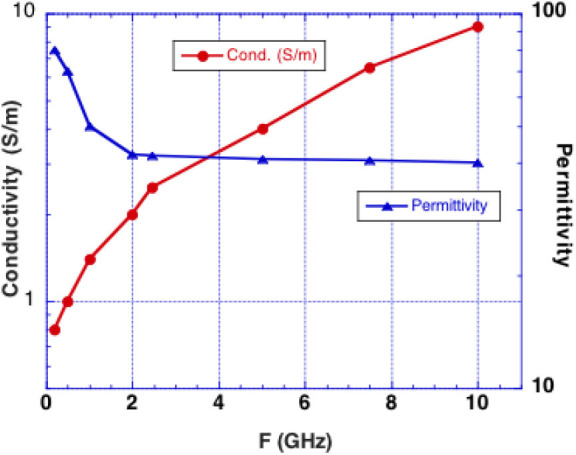
Experimentally determined conductivity and permittivity of white and gray matter vs. RF frequency (19). Conductivity is used to calculate the penetration depth into brain tissue (called skin depth in electromagnetic nomenclature). Permittivity is used to calculate the microwave wavelength in brain tissue.

**Figure 2:**
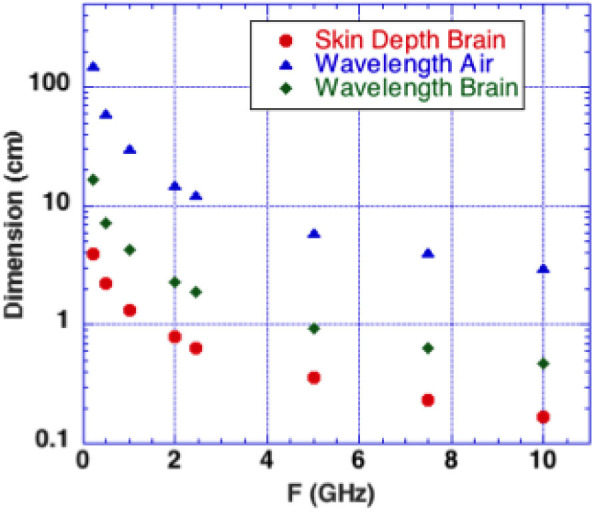
Microwaves in air and in brain vs. microwave frequency. Calculated depth of penetration of microwaves into the brain is shown. Energy is reduced by a factor 1/2.7 at a superficial level termed “skin depth” using electromagnetic nomenclature.

We consider microwave frequency ranges and wavelengths from 300 MHz (1 m) to 10 GHz (3 cm) in air where availability of microwave sources coherent on short time scales (i.e., 50 μs) exist. The permittivity and conductivity of white and gray brain matter are shown in Figure 1 (20). Figure 2 shows the depth into brain tissue where the microwave energy is ~1/2.7 of incident energy. Note that microwave wavelengths in air and brain tissue are functions of microwave frequency. The microwave wavelengths in brain tissue range from 0.5 to 18 cm with 1/2.7 attenuation depths of 0.2–4 cm. The dominant interaction of 1–10 GHz microwave frequencies in water relate to absorption at a “Debye” peak at these lower microwave frequencies related to migration defects through the H bond water network (21).

Microwave interactions with the human head were early described by Frey et al. (22) (first reported microwave effects upon the auditory system). His detailed descriptions were designated as the *Frey Effec*t (22). Subsequently, Lin et al. (23) clarified the fact that square microwave pulses are audible. Experimental modeling determined that the microwave pulse rapidly heats tissue in the “skin” brain depth (depth of 1/2.7 of incident energy). Figure 2 shows that 0.3–10 GHz microwave radiation penetrates a few cm to a few mm into brain tissue. The resulting thermal expansion may launch an acoustic wave by thermoelastic effect traveling by bone conduction to the inner ear where it activates cochlear receptors (23). A single microwave pulse may thus be perceived as an acoustic click while a train of microwave pulses is sensed as an audible tone with a pitch corresponding to the pulse repetition rate.

Watanabe et al. (24) employed finite differential analysis to model the effect of 1 mW/cm^2^, 915 MHz single 20 μs wide square pulses (rise time 400 ns) incident upon the back of realistic human head models. These workers found that thermoelastic coupling of microwave energy into the brain occurred near the brain surface, launching an acoustic wave propagating to the opposite side of the head at the speed of sound in water and reverberating up to several times. Reverberation frequencies were found to range from 7 to 9 kHz as determined by the transit times across a 14 cm skull cavity. Use of a 50 μs pulse length with a repetition rate of 7–9 kHz maximized energy coupling to brain tissue (24). Longer pulses or higher repetition rates produced destructive interferences that canceled some of the impinging microwave energy. Other than the skin depth dependence, this mechanism may be similar for any microwave frequency in the 0.3–10 GHz range, dependent mainly on the frequency with which the microwave pulses are delivered.

We now present three other possible head-microwave interactions not previously considered.

**Figure 3:**
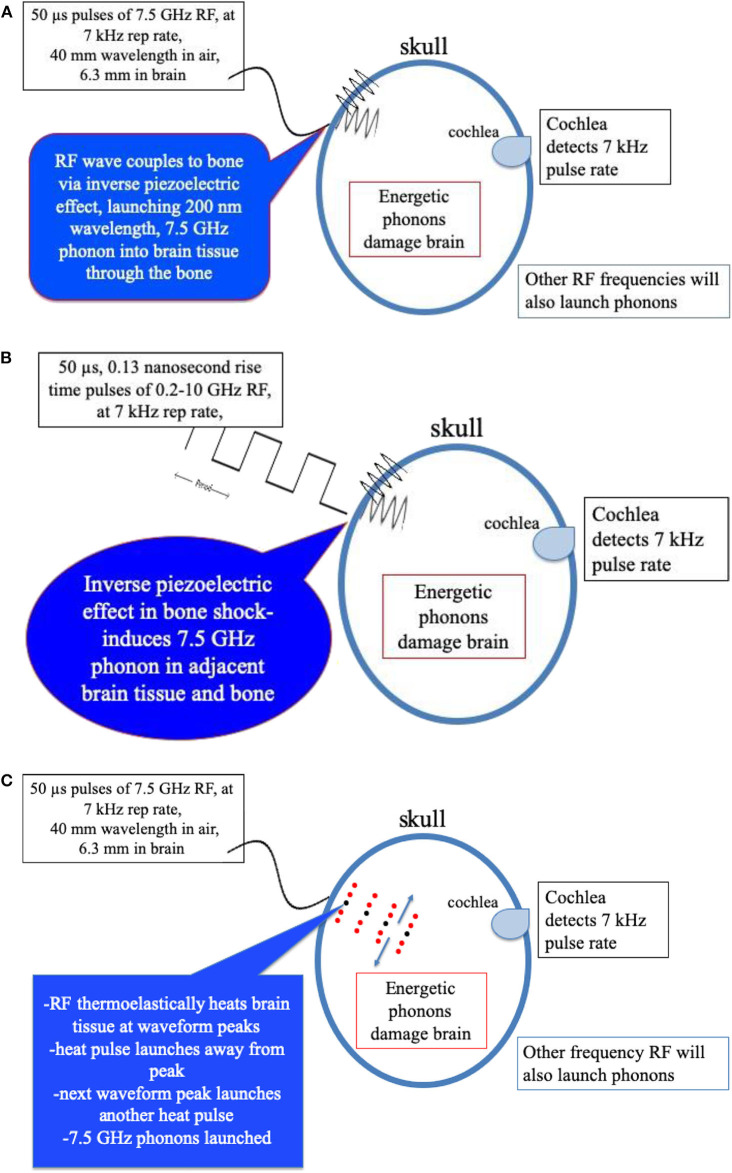
**(A)** Mechanism of bone peizoelectric effects in launching phonons in brain tissue water. **(B)** Schematic representation of transduction of fast rise-time pulsed microwaves to acoustic phonons in brain tissue through the shock response of the *inverse* piezoelectric effect in the bone of the skull. **(C)** Schematic representation of transduction of pulsed microwaves to acoustic waves in brain through the thermoelectric effect in brain tissue water.

1. Wieland et al. (25) employed a cyclotron x-ray light source and x-ray diffraction to measure actual displacements in bovine bone samples. They detected strains as small as 8 × 10^−6^ due to the resulting inverse piezoelectric effect which induces strain due to applied electric field (25). Strains as large as 9 × 10^−4^ were detected with electric field exposures of ~6,000 V/m or ~6 V/mm. Amplitude of microwave exposure of 1 volt/mm resulted in a sizable strain of 1.5 × 10^4^. Measurements of bone dielectric properties indicate that molecules in bone also respond to low GHz radiation values (20)^1^. Sufficiently large microwave power could send energy to the ear directly through the bone sensed by affected individuals as painful and damaging to hearing. Pulsed microwave energy may also launch acoustic waves into the brain tissue adjacent to the skull at the same frequency. Figure 3A shows a schematic representation of this possible mechanism of bone piezoelectric effects coupling microwave energy into phonons in brain tissue through piezoelectric response of skull bone.
2. A shock wave created by a sudden strain induced in bone of the skull is another possible transduction mechanism inducing launch of acoustic phonons in brain water. Here, the pulse rise time of the microwaves may be an important parameter. If, for example, the rise time of the microwave pulse is as rapid as (7.5 GHz)^−1^ or 0.13 ns, this level of acoustic shock could excite the lowest acoustic phonon in water with a 7.5 GHz frequency (13–15). With sufficient power, such energy could initiate a damage mechanism as occurs in explosive shock with skull bone rippling, Figure 3B schematically depicts this effect.
3. While electromagnetic radiation is known to transduce to optical phonons, electromagnetic radiation was not earlier believed capable of acoustic phonon coupling. However, Nelson et al. (26), using “Laser Induced Phonons” (LIPS) methodology, demonstrated that electromagnetic radiation is capable of coupling to acoustic phonons in absorbing liquids. These investigators used two ~532 nm lasers with slightly different wavelengths to produce a difference interference wavelength tunable in the 1–30 GHz region by tuning one of the lasers. The absorbed light heats the liquid at the peaks of the differing laser wavelength, causing thermal expansion, thus launching acoustic waves at that specific wavelength. A secondary laser probe diffracted by the momentary diffraction grating generated by the peaks of the acoustic waves then detects resulting acoustic phonons within the liquid. Figure 3C is a schematic representation of non-uniform heating phenomena capable of transducing microwave energy into acoustic phonon waves in brain tissue.

Experimental laser diffraction grating effects do not disappear immediately after 100 ps (ps) excitation pulses end. The effect persists for many microseconds, suggesting that short (~ps) relaxation times of rotational states cause water to sustain large spatial temperature gradients for relatively long-time intervals (21). This effect implies that non-uniform heating of water could also launch high frequency acoustic waves. Thus, a third possible mechanism coupling microwave energy into acoustic phonons may be rapid heating of water in the brain tissue at the peaks of the microwave waveforms. In this case, microwaves heat water directly rather than by the interference effects of two optical wavelength lasers. The net end results of phonon generation appear to be similar.

**Figure 4:**
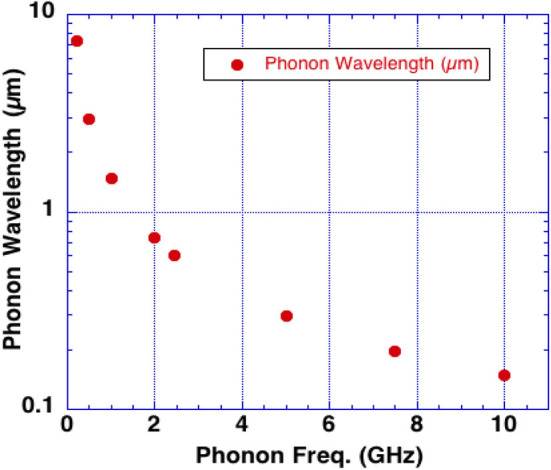
Phonon wavelengths in water vs. phonon frequency (wavelength = sound velocity/frequency).

Rapid heating can cause thermal expansion (the thermoelastic effect) exciting acoustic waves in water at the frequency of the incident microwaves. Brain tissue may be especially susceptible to excitation of an intrinsic acoustic phonon at a frequency of 7.5 GHz due to its increased lifetime as compared to other frequencies. Figure 4 shows the phonon wavelengths in water vs. phonon frequency. Note the phonon effects are also active at these lower wavelengths.

Microwave pulses or short rise time pulses may therefore excite phonons by several mechanisms. We hypothesize that, (i) the inverse piezoelectric effect in the skull, (ii) fast rise-time shock, (iii) and microwave absorption in water in the brain are all capable of launching acoustic waves that produce the sounds heard by targeted subjects through the Frey Effect (22). With sufficient energy input, brain damage likely occurs by a phonon energy mechanisms exceeding brain tissue strength (13, 14). The efficacy of these mechanisms to produce brain damage also depends upon microwave frequency as shown in Figure 4 along with microwave pulse rise-time.

## Discussion

Incident durations described by injured personnel are as follows: “*The sound seemed to manifest in pulses of varying lengths – seven seconds, 12 seconds, two seconds – with some sustained periods of several minutes or more. Then there would be silence for a second, or 13 seconds, or four seconds, before the sound abruptly started again”* (5). The repetition rate from the AP news report (5) claimed to be a central frequency of 7,266 Hz with several frequencies spaced 200 Hz on either side of 7,266 Hz. The microwave frequency within the pulses and the pulse width of the microwaves triggering the audible effect remain unknown.

Igarashi et al. (27) showed 50% mortality with extensive gross brain damage in rats directly exposed at close range to a single high pulse of 3 kW, 2.45 GHz microwaves for 0.1 s. Based upon the size of the rats and the microwave horn used, we estimate the incident power density to be ~1 kW/cm^2^ which would deliver to the target an average power of 1,000 W/cm^2^ (27). By contrast, a 30-min application of 2.8 GHz pulses using power as low as 15-mWcm^−2^ was seen to damage rat brains (10). In the absence of known experimental threshold power inducing brain damage, we suggest initially beginning with a minimum average power delivered in a focused microwave beam to ~1 Wcm^2^. For a 7 kHz repetition rate using 50 μs pulses, the individual pulse power at target would be ~1 Wcm^−2^/duty cycle = ~3 Wcm^−2^. These estimated values are useful stating points for empiric experimental observations.

Lin et al. (6, 7) suggested that pulsed microwaves were the likely means by which the U.S. Cuban embassy personnel were injured. However, the precise mechanisms by which microwaves cause brain injury require delineation. To reiterate, we propose that microwaves can transduce acoustic waves in brain water by three possible mechanisms: (i) inverse bone piezoelectric effects, (ii) fast pulse rise-time shock affecting bone, and (iii) thermoelastic absorption, at GHz frequencies. We present hypotheses on how pulsed microwave transduced acoustic waves from a directed energy beam with specific characteristics induce nanometer scale intracellular brain damage. Such damage is likely, as with low intensity blast, best detected by TEM (18, 19). Clinical detection of imaging abnormalities requires use of DTI, a water imaging technique. The hypothesis that dimensions of injury due to microwave brain tissue may be similar to that of coupling of low intensity primary shock wave energy causing blast-induced (mTBI) requires experimental verification. The commonality of dimensions of phonon excitation may account for these dimensional similarities. Later symptoms displayed by embassy personnel also appear to mimic injury characteristics of mTBI caused by primary low-level blast (1–3). In addition to presenting a consistent physical nanoscale model of brain damage, the present working hypothesis may also explain why pulsed microwaves are more damaging than continuous wave microwaves. Threshold characteristics of differing pulsed wave microwave energies damage remain to be determined.

Microwave exposure alters blood brain barrier permeability to cause DNA damage (11, 12). Blood brain injury also is known to occur in blast injury but is much better described (28–30). A critical review by Zhi et al. (31) concluded that animal studies remain conflicting and inconclusive. The magnitude of the microwave energetics under consideration, compared to high level blast effects causing hollow organ and lung damage, is orders of magnitude less. Energies involved in this injury compare to those found in low level blast effects ranging from ~47 kPa to levels <100 kPa resulting in nanoscale injuries in the absence of gross or microscopic organ damage (18). Long term studies of single or pulsed microwave injuries resulting in chronic glial or astrocytic effects have not been done, while limited data on blood brain barrier effects imply endothelial vacuolization in the olfactory region (11, 12). High explosive blasts have long been known to generate wide wavelength microwaves likely with differing effects than the short-wave high frequency microwaves here considered (32).

Further studies of apparently conflicting data are required (31). Postulated microwave brain damage has yet to be fully characterized experimentally. The present analysis of injury mechanisms is based upon well-founded physical principles and observations. Exploration of exposure times, power and specific microwave wavelengths here considered can serve to define dimensions of microwave brain injury, optimal diagnostic methods, and eventual protective measures. Limitations of the present theory include the need to postulate three hypotheses possibly inducing phonon generation in water. These alternative possibilities suggest several approaches to experimental exploration.

We here have presented physical theory, injury hypotheses and biological findings related to microwave brain damage. These injuries can be explored by exposure of animal models to varying microwave exposure times, power, frequencies, and pulses including magnitude and frequency as compared to controls. Subsequent neurobehavioral testing followed by comprehensive examination of brain tissues including TEM will be needed to uncover ultrastructural damage. Parameters of microwave power thresholds, frequency, duration and pulse characteristics causing specific types of brain injury require varying types of experimental exposures. An initial starting point of 7 kHz repetition rate using 50 μs pulses, the individual pulse power at a murine target of ~1 Wcm^−2^/duty cycle = ~3 Wcm^−2^ is suggested. Microwave skull interactions require exploration using pulsed microwave exposure of diploic skull bone or piezoelectric bone surrogates *in vitro* adjacent to 0.9% isotonic saline. High frequency transducers attached to bone and in adjacent water can be used detect high frequency transduced acoustic waves. Thermoelastic phonon generation mechanism in normal saline solution alone could also be explored using direct microwave exposure to study basic aspects *in vitro* phonon generation in water. Data thus obtained *in vitro* can be used to guide *initial* specific parameters of power, wavelength and pulse frequency likely to cause *in vivo* microwave brain injury.

## Data Availability Statement

The raw data supporting the conclusions of this article will be made available by the authors, without undue reservation.

## Author Contributions

All authors listed have made a substantial, direct and intellectual contribution to the work, and approved it for publication.

## Conflict of Interest

The authors declare that the research was conducted in the absence of any commercial or financial relationships that could be construed as a potential conflict of interest.

## Footnotes

^1^Most crystals exhibit THz phonon frequencies. Thus, atoms in crystals will be displaced by the inverse piezoelectric effect at 1–10 GHz frequencies with relatively low efficiency.
